# NF-κB transcriptional activation by TNFα requires phospholipase C, extracellular signal-regulated kinase 2 and poly(ADP-ribose) polymerase-1

**DOI:** 10.1186/s12974-015-0448-8

**Published:** 2015-12-04

**Authors:** Billy Vuong, Adam D. J. Hogan-Cann, Conrad C. Alano, Mackenzie Stevenson, Wai Yee Chan, Christopher M. Anderson, Raymond A. Swanson, Tiina M. Kauppinen

**Affiliations:** Department of Pharmacology and Therapeutics, Faculty of Health Sciences, University of Manitoba, 753 McDermot Avenue, Winnipeg, MB R3E 0T6 Canada; Neuroscience Research Program, Health Sciences Centre and College of Medicine, Kleysen Institute for Advanced Medicine, 710 William Avenue, Winnipeg, MB R3E 0Z3 Canada; Department of Neurology, University of California San Francisco, and Veterans Affairs Medical Center, 4150 Clement Street, San Francisco, CA 94121 USA; Present Address: Genentech Inc., 1 DNA Way, South San Francisco, CA 94080 USA

**Keywords:** Astrocyte, Calcium, DNA damage, ERK2, Inflammation, Microglia, NF-κB, PARP-1, PLC, TNFα

## Abstract

**Background:**

The nuclear enzyme poly(ADP-ribose) polymerase-1 (PARP-1) is required for pro-inflammatory effects of TNFα. Our previous studies demonstrated that PARP-1 mediates TNFα-induced NF-κB activation in glia. Here, we evaluated the mechanisms by which TNFα activates PARP-1 and PARP-1 mediates NF-κB activation.

**Methods:**

Primary cultures of mouse cortical astrocytes and microglia were treated with TNFα and suitable signaling pathway modulators (pharmacological and molecular). Outcome measures included calcium imaging, PARP-1 activation status, NF-κB transcriptional activity, DNA damage assesment and cytokine relesease profiling.

**Results:**

TNFα induces PARP-1 activation in the absence of detectable DNA strand breaks, as measured by the PANT assay. TNFα-induced transcriptional activation of NF-κB requires PARP-1 enzymatic activity. Enzymatic activation of PARP-1 by TNFα was blocked in Ca^2+^-free medium, by Ca^2+^ chelation with BAPTA-AM, and by D609, an inhibitor of phoshatidyl choline-specific phospholipase C (PC-PLC), but not by thapsigargin or by U73112, an inhibitor of phosphatidyl inisitol-specific PLC (PI -PLC). A TNFR1 blocking antibody reduced Ca^2+^ influx and PARP-1 activation. TNFα-induced PARP-1 activation was also blocked by siRNA downregulation of ERK2 and by PD98059, an inhibitor of the MEK / ERK protein kinase cascade. Moreover, TNFα-induced NF-κB (p65) transcriptional activation was absent in cells expressing PARP-1 that lacked ERK2 phosphorylation sites, while basal NF-κB transcriptional activation increased in cells expressing PARP-1 with a phosphomimetic substitution at an ERK2 phophorylation site.

**Conclusions:**

These results suggest that TNFα induces PARP-1 activation through a signaling pathway involving TNFR1, Ca^2+^ influx, activation of PC-PLC, and activation of the MEK1 / ERK2 protein kinase cascade. TNFα-induced PARP-1 activation is not associated with DNA damage, but ERK2 mediated phosphorylation of PARP-1.

## Background

Tumor necrosis factor alpha (TNFα) has pro-inflammatory effects in the nervous system and other tissues. These effects are due in part to its ability to induce nuclear factor kappa B (NF-κB) activation (as reviewed by [1, 2]). The high-affinity TNFα receptors, TNFR1 and TNFR2, are both expressed on astrocytes and microglia of the central nervous system [3, 4], and TNFα binding to either of these receptors initiates signaling cascades that activate the IKK kinase complex [1, 2, 5]. In the canonical NF-κB signaling pathway, the IKK kinase complex phophorylates the IκB subunit of cytosolic NF-κB complex, leading to the release and nuclear translocation of NF-κB dimers. These NF-κB dimers bind to κB sites in promoter regions of target genes, including many genes encoding pro-inflammatory proteins (as reviewed by [2, 5]). However, nuclear translocation and DNA binding by NF-κB is not sufficient to induce gene transcription. This requires for NF-κB to form a functional transcription complex with other proteins. Evidence suggests that poly(ADP-ribose) polymerase-1 (PARP-1) is a key component of the NF-κB transcription complex [6–8]. PARP-1 forms branched poly(ADP) ribose (PAR) polymers on several proteins involved in DNA repair and transcription, including PARP-1 itself (PARP-1 automodification). In addition, many nuclear proteins express binding sites for these PAR polymers [9, 10]. The protein-protein interactions resulting from PAR formation and PAR binding modulate the function of several transcription factors, including NF-κB [6, 11, 12], thus providing a link between PARP-1 and cellular inflammatory responses. PARP-1 enzymatic inhibitors can suppress inflammatory responses in microglia [11, 13–15] and other cell types [12, 13, 16–21]. However, it has also been reported that enzymatically inactive PARP-1 can facilitate NF-κB transcriptional activation [22–24], suggesting that the anti-inflammatory effects of PARP-1 inhibitors might occur by a different mechanism.

The signal transduction pathway leading from TNFα receptor activation to PARP-1 activation has not been established. PARP-1 activation is classically induced by DNA damage, in which PARP-1 binding to DNA strand breaks or kinks triggers enzymatic activation and poly(ADP-ribosyl)ation of histones and other proteins associated with DNA repair [22]. PARP-1 was recently shown to also be activated under several conditions in which DNA damage is not known to occur, including DNA transcription, DNA replication, and in neurons, neurotrophin responses and memory consolidation [25–29]. Studies suggest that ERK2 activation and release of endoplasmic reticulum Ca^2+^ stores are involved in DNA damage independent of PARP-1 activation in neurons [30, 31], but little is known about this signaling pathway in inflammatory cells or TNFα signaling.

Goals of the present study were to establish the mechanism by which TNFα leads to PARP-1 activation and to determine whether inhibition of PARP-1 enzymatic activity blocks TNFα-induced transcriptional activation of NF-κB. These studies used primary cell cultures of microglia and astrocytes in order to avoid artifacts inherent in the use of immortalized cell lines. Microglia are resident immune cells of the central nervous system, and astrocytes are glial cells that contribute to the inflammatory response in brain. Results of these studies show that TNFα leads to PARP-1 activation by a pathway independent of DNA damage, involving phosphatidyl choline-specific phospholipase C (PC-PLC), and ERK. The present study also demonstrated that inhibition of this pathway and inhibition of PARP-1 enzymatic activity both prevent NF-κB-mediated gene transcription.

## Methods

### Materials

PARP-1 inhibitors, 3,4-dihydro-5-[4-(1-piperidinyl)butoxy]-1(2 h)-isoquinolinone (DPQ) and *N*-(6-oxo-5,6-dihydrophenanthridin-2-yl)-*N*,*N*-dimethylacetamide (PJ34) were obtained from Calbiochem (San Diego, CA) and Inotek Pharmaceuticals (Lexington, MA), respectively. The MAPK inhibitor 2-(2-amino-3-methoxyphenyl)-4H-1-benzopyran-4-one (PD98059) was obtained from Tocris Cookson Ltd. (Ellisville, MO). The PLC activator, 2,4,6-trimethyl-N-[3-(trifluoromethyl)phenyl]benzenesul fonamide (m-3M3FBS) and PLC inhibitors, 1-[6-((17β-3-methoxyestra-1,3,5(10)-trien-17-yl)amino)hexyl]-1H-pyrrole-2,5-dione (U-73112) and tricyclodecane-9-yl-xanthogenate (D609) were obtained from Calbiochem. Cell culture reagents were obtained from Cellgro/Mediatech (Herndon,VI), and all other reagents were from Sigma-Aldrich (St. Louis, MO) except where otherwise stated.

### Cell cultures

All animal studies were approved by the San Francisco Veterans Affairs Medical Center animal studies committee and University of Manitoba animal care and use committee, and follow the NIH guidelines for humane care of animals. Cultures were prepared from PARP-1^−/−^, and wild-type (wt) mice as described previously [14]. The PARP-1^−/−^ mice were descendants of the 129S-Adprtl^tmlZqw^ strain, originally developed by Wang et al. [32] and obtained from the Jackson Laboratory (Bar Harbor, ME). These mice were outbred for seven generations with wt CD-1 mice, and wt CD-1 mice were used as controls for the PARP-1^−/−^ mice. Astrocyte cultures were prepared from 1-day old mouse pups of both sexes in 24-well plates as described previously [33]. At confluency, microglia were harvested by gently shaking and re-plated at density of 5 × 10^5^ cells/well on a 24-well plate [14]. The microglia cultures were subsequently maintained in glia-conditioned medium. The confluent astrocyte cultures were treated with 22 μM cytosine β-D-arabinofuranoside for 2 days to inhibit the proliferation of remaining microglia. The astrocyte cultures were subsequently maintained in Eagle’s minimal essential medium (MEM) supplemented with 3 % fetal bovine serum (FBS).

### Drug incubations

Studies using astrocyte cultures were initiated by replacing the culture medium with a physiologically balanced salt solution (BSS) containing 3.1 mM KCl, 134 mM NaCl, 1.2 mM CaCl_2_, 1.2 mM MgSO_4_, 0.5 mM KH_2_PO_4_, 15.7 mM NaHCO_3_, and 2 mM glucose, pre-equilibrated to pH 7.2 in a 5 % CO_2_ atmosphere. Drugs were prepared as concentrated stock solutions in BSS. Experiments were performed at 37 °C in a 5 % CO_2_ atmosphere and were terminated by complete medium exchange and replacement with fresh BSS. Studies with microglial cultures were performed identically but used MEM rather than BSS.

### Immunostaining

Fixation and immunostaining of cell cultures was performed as previously described [14]. For detection of poly(ADP-ribose), incubations were performed with a 1:1000 dilution of rabbit anti-poly(ADP-ribose) (Trevigen, Gaithersburg, MD, Cat# 4336-BPC) at 4 °C for 24 h, followed by incubation with Alexa Fluor 594-conjugated anti-rabbit IgG (Molecular Probes, Eugene, OR), 1:500 dilution, for 2 h at room temperature. For evaluation of NF-κB p65 subunit translocation, incubations were performed with a 1:30 dilution of rabbit polyclonal anti- p65 (Cell Signaling Technology, Inc., Danvers, MA, Cat# 3987) at 4 °C for 24 h, followed by incubation with Alexa Fluor 594-conjugated anti-rabbit IgG (Molecular Probes, Eugene, OR), 1:500 dilution for 2 h. In some studies, the cells were subsequently incubated with 2 μg/ml propidium iodide for 5 min to obtain nuclear counterstaining. Confocal photomicrographs of astrocytes were obtained with a Zeiss LSM 510 META with a dual-PMT detector and a 32-channel META detector and Zeiss AIM imaging software. Microglia images were obtained with standard epifluorescence microscopy because these cells could not be cultured in a resting state on glass coverslips. Controls prepared in the absence of primary antibody showed no staining under the conditions described (not shown). DNA strand breaks were detected by DNA-polymerase I-mediated biotin-dATP nick translation (PANT) [34]. Cultures were permeabilized in 1 % Triton X-100 in phosphate buffered saline (PBS) for 30 min, and endogenous peroxidase was quenched with 2 % hydrogen peroxide. The cultures were then incubated for 90 min with the PANT reaction mixture: 5 mM MgCl2, 10 mM 2-mercaptoethanol, 20 μg/ml bovine serum albumin, 30 μM dGTP, 30 μM dCTP, 30 μM dTTP, 1 μM dATP, 29 μM biotinylated dATP, and 40 U/ml of DNA-polymerase I in PBS, pH 7.4. The biotinylated areas of DNA strand breaks were visualized by subsequent incubation with streptavidin-horseradish peroxidase and DAB peroxidase substrate. Controls incubated with PANT reaction mixture without DNA-polymerase I showed no staining.

### siRNA downregulation of ERK1/2

Astrocytes were incubated with siRNA transfection complexes at day 10 in vitro, at which time they are 95 % confluent. siRNA incubations were performed with RNAiFect reagents (Qiagen Inc., CA) according to the manufacturer’s instructions and as previously described [35]. The culture medium of each well was replaced with 400 μl of transfection complex mixture containing 1 μg of ERK1 siRNA or 3 μg of ERK2 siRNA and 6 μl of RNAiFect in optiMEM. The siRNA sense sequence for ERK1 was 5′ ACAAGCGCAUCACAGUAGAtt 3′ and for ERK2 was 5′ CAAAGUUCGAGUUGCUAUCtt 3′ (Ambion Inc, TX, USA). Controls were prepared with 3 μg of a mismatch sequence lacking significant homology to any known mouse gene sequences. The siRNA complexes were removed after 6 h and replaced with culture medium. Four days later when cultures were used for experiments, the percentage of siRNA-induced reduction in ERK1 and ERK2 expressions was determined by quantitating the ERK band size in ERK siRNA vs. mismatch siRNA transfected cultures in both ctrl and TNFα conditions.

### Lentivirus NF-κB reporter gene transfection

The lentivirus NF-κB reporter gene construct (pLenti-kB-dEGFP) has a destabilized enhanced green fluorescent protein (dEGFP) construct under the control of five tandem repeats of κB-enhancer elements specific for p65and a plain TATA box [36]. Transfections were performed as previously described [15]. Astrocytes were infected with the Lenti-κB-dEGFP 4–5 days before experiments. EGFP expression was evaluated 1 and 24 h after exposure to TNFα by counting EGFP-expressing cells from five random fields within each culture well.

### TNFR1 inhibition

Cells were incubated with 100 μg/ml of TNFR1 neutralizing antibody (anti-mouse CD120a, Affymetrix eBioscience, San Diego, CA, Cat#16-1202) or control IgG (Armenian hamster IgG, Affymetrix eBioscience, San Diego, CA, Cat#16-4888) in MEM for 4 h prior to starting the experiments.

### Calcium imaging

Intracellular calcium levels were measured in astrocytes loaded with Fura-2-AM (Molecular Probes; 3 μM, 60 min). Experiments were carried out in bicarbonate-free BSS at 37 °C and buffered to pH 7.2 with 10 mM PIPES. Calcium-free BSS was prepared by omitting CaCl_2_ and adding 2 mM EGTA. The change in dye fluorescence (Fura 340/380 ratio) was measured with aZeiss 200-M inverted microscope outfitted with an ORCA ER II microscope system using Openlab Improvision software. Excitation and emission wavelengths were controlled by Sutter filterwheels and shutters. Baseline values were recorded for 15–20 min prior to drug additions. At the end of each experiment, the calcium ionophore A23187-Br (10 μM) was applied to calibrate the intracellular signal. Data are presented as a change in Fura2 emission (Fura 340/380 ratio), and as a mean change in Fura 340/380 ratio during 15-, 30-, and 60-min intervals from three to four independent experiments. In each independent experiment data were acquired from six to ten cells at 90-s intervals, and at 10-s intervals in neutralizing antibody experiments. The high-affinity dye, Fura-2, was used to maximize sensitivity at low intracellular calcium concentrations, and therefore, the signal is likely saturated and underestimates the differences between Ca^2+^-free and Ca^2+^-containing medium.

### Western blotting

Western blots were prepared and quantified as described [14]. Membranes were incubated with 1:1000 dilutions of rabbit anti-ERK1/2 or anti phospho-ERK1/2 (Cell Signaling Technology, Beverly, MA, Cat# 9102 and 4376), or with a 1:500 dilution of mouse monoclonal anti-poly(ADP-ribose) (Trevigen, Gaithersburg, MD, Cat# 4335-MC). After washing, the membranes were incubated for 2 h with peroxidase-conjugated anti-rabbit or anti-mouse IgG (Vector Laboratories, Burlingame, CA) diluted 1:7500. The protein bands were visualized using ECL^TM^ WB Detection kit (Amersham-Pharmacia Biotech) and X-OMAT AR film (Kodak) or ChemiDoc MP imaging system (BioRad). To quantify protein loading, the membranes were re-probed with mouse monoclonal anti β-actin at a 1:10,000 dilutions, followed by peroxidase-conjugated anti-mouse IgG (1:10,000 dilutions). Controls performed in the absence of primary or secondary antibodies showed no signal (data not shown). Band densities were quantified with the NIH Image J program.

### Site-directed PARP-1 mutations

The mutant PARP-1 constructs were prepared as described [35]. In short, human PARP-1 cDNA (hPARP-1, BC03754 from NIH Mammalian Gene Collection) was corrected to match the human genomic sequence at amino acid residue 762 (from alanine to valine) using Site-Directed Mutagenesis kit (Stratagene). Single (S372E) or double mutants (S372A and T373A) of these constructs were then generated and sequence-verified. PARP-1^−/−^ astrocytes were transfected 2 days before experiments with the PARP-1 constructs by replacing the culture medium with 300 μl of transfection complex mixture containing 0.5 μg of DNA and 1 μl of Lipofectamine 2000 (Invitrogen) in OptiMEM. The medium was replaced with culture medium 8 h after the initiation of transfection and cells were used for experiments 40 h later. The transfection efficacy of this method has been established previously by western blots detecting PARP-1 expression with a polyclonal antibody (ALX-210-302, Alexis/Enzo Life Sciences, Plymounth Meeting, PA) [35].

### Cytokine assay

Cytokines were analyzed in 50-μl aliquots of cell culture medium using a Milliplex mouse multiplex immunoassay bead system according to the manufacturer’s instructions (Millipore) [37]. Samples were assayed in duplicate, and the fluorescent signal corresponding to each cytokine was measured with a BioPlex 200 system (BioRad, Hercules, CA) in parallel with known standards. Cytokine concentrations measured from culture medium were normalized to the protein content of each well as determined by the bicinchoninic assay [38].

### Statistics

For cell culture studies, each “n” denotes an independent experiment, where each experiment is from a separate cell culture preparation and comprised of 3–4 parallel treatments per condition. Results are presented as a means ± standard error. Statistical significance was evaluated by one-way ANOVA followed by the Student-Newman-Keuls multiple comparison test. *p* values below 0.05 were considered significant.

## Results

### TNFα-induced PARP-1 activation in the absence of detectable DNA damage

**Fig. 1:**
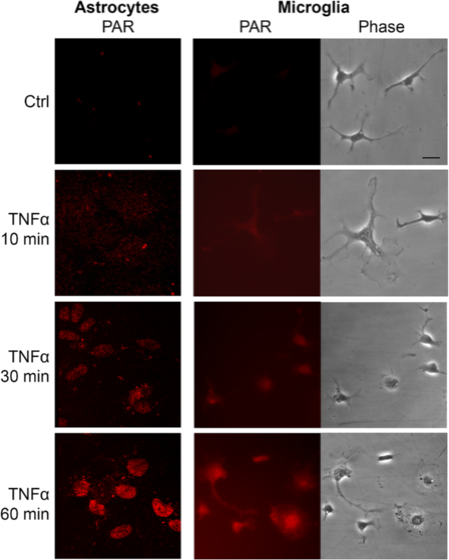
TNFα induces PARP-1 activation in microglia and astrocytes. Immunostaining for poly(ADP-ribose) (labeled as PAR) shows accumulation in both astrocytes and microglia during incubation with TNFα (15 ng/ml). Phase contrast images shows simultaneous morphological transformation of microglia. Astrocytes do not exhibit morphological changes (not shown). *Scale bar*, 10 μm. Representative of *n* = 4

TNFα at physiological concentration (15 ng/ml) induced a rapid accumulation of PAR polymers, a product of PARP-1 enzymatic activity, in both astrocyte and microglial cultures (Fig. 1). PAR formation in microglia was time-linked to morphological activation, as characterized by process retraction and soma enlargement (Fig. 1). Astrocytes, unlike microglia, do not undergo morphological changes in response to TNFα (not shown). Prior studies have shown that TNFα does not induce PAR accumulation in PARP-1^−/−^ cells [14], indicating that PARP-1 is the major source of PAR formation [15, 39, 40].

**Fig. 2:**
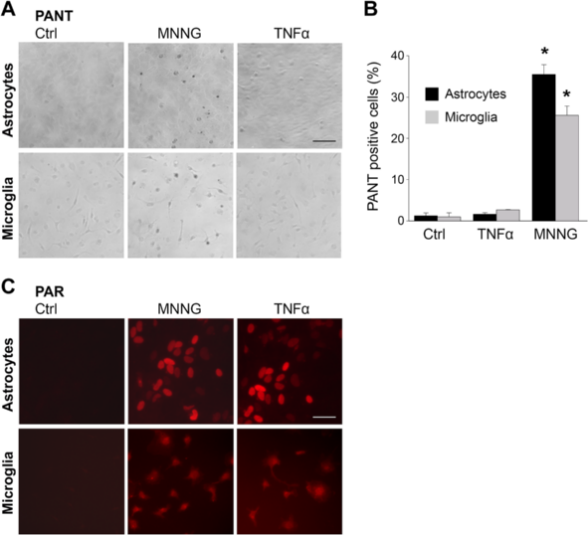
TNFα does not induce detectable DNA strand breaks. **a** PANT staining shows no detectable increase in DNA strand breaks in either microglia or astrocyte cultures after 60-min incubation with 15 ng/ml TNFα. The DNA alkylating agent, MNNG (10 μM) is used as a positive control. *Scale bar*, 50 μm. **b** Quantification of PANT positive cells (* *p* < 0.05, *n* = 3). **c** Immunostaining for PAR shows comparable PAR accumulation in cells incubated for 60 min with 15 ng/ml TNFα or 10 μM MNNG. *Scale bar*, 25 μm

PARP-1 activation is classically induced by DNA damage, particularly DNA strand breaks [22, 41]. To determine if DNA damage was involved in TNFα-induced PARP-1 activation, cultures were evaluated with the DNA-polymerase I-mediated biotin-dATP nick translation (PANT) technique for single-strand DNA breaks. Cultures fixed after 1 h of TNFα incubation, a time point with robust PAR accumulation, showed no increase in PANT staining. By contrast, an extensive PANT signaling was observed in cells treated with 10 μM N-methyl-N'-nitro-N-nitrosoguanidine (MNNG), a DNA alkylating agent widely used to induce PARP-1 activation [42]. The degree of PARP-1 activation induced by this concentration of MNNG was comparable to that observed in cultures treated with TNFα (Fig. 2c).

### TNFα induces PARP-1 activation through a signaling pathway involving PLC and Ca^2+^

**Fig. 3:**
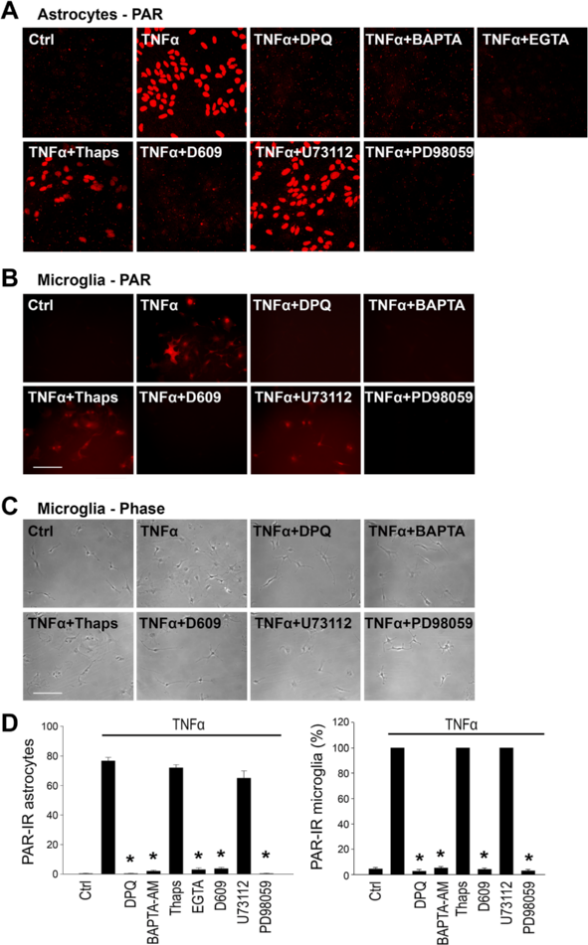
TNFα-induced PARP-1 activation requires PLC, Ca^2+^ influx, and activation of MAPK-ERK kinase. **a** Astrocyte cultures were immunostained for PAR after 60-min incubations with 15 ng/ml TNFα. PAR formation was prevented in calcium-free medium (containing 500 μM EGTA) and by the calcium chelator BAPTA-AM (2 μM), but not by pre-treatment with thapsigargin (2 μM for 15 min). PAR formation was also prevented by the PC-PLC inhibitor, D609 (10 μM), and by the MAPK-ERK inhibitor, PD98059 (10 μM). The PI-PLC inhibitor U-73112 (10 μM) did not block PAR formation. An absent signal in cultures treated with the PARP inhibitor DPQ (25 μM) confirms the specificity of the immunostaining. Representative of *n* = 4. **b** PAR immunostaining in microglia shows the same pattern as observed in astrocytes. Studies using EGTA were not possible with microglia cultures due to rapid cell detachment. **c** Phase contrast images of same microglial files shown in **b**. Microglial morphological transformation induced by TNFα is absent under the conditions in which PAR formation was blocked. *Scale bars*, 50 μm. **d** Quantification of PAR positive cells (avarage number per field in confluent astoglia cultures; percent of positive cells in microglia cultures). (*p < 0.05, *n* = 3–4)

**Fig. 4:**
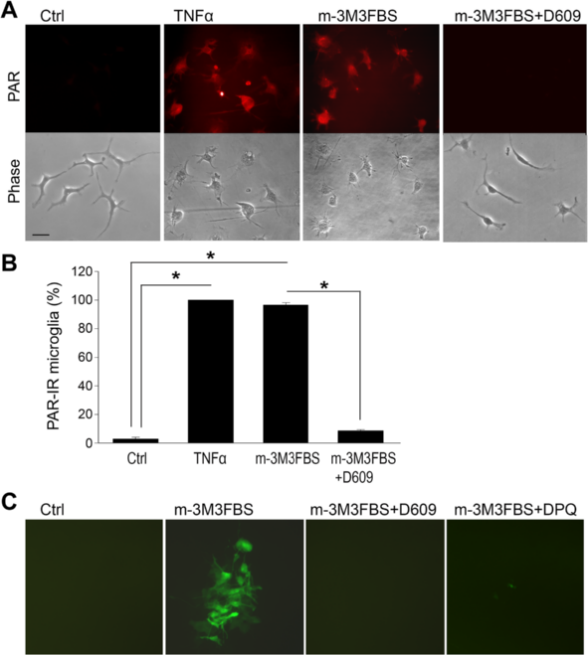
PLC activation triggers microglial morphologial transformation and activation of PARP and NF-κB, independent of TNFα. **a** The PLC activator m-3M3FBS (25 μM, 60 min) induces microglial morphological transformation and PARP activation. The PC-PLC inhibitor, D609 (10 μM) prevents both effects of the PLC activator. *Scale bar*, 10 μm. **b** Quantification of PAR positive cells (*p < 0.05, *n* = 3). **c** NF-κB transcriptional activity was evaluated in cultures transfected with a reporter gene in which dEGFP expression (*green*) is driven by NF-κB p65 subunit binding. The PLC activator m-3M3FBS (25 μM, 60 min) induced NF-κB activation as seen by dEGFP expression. This NF-κB transcriptional activity was blocked by both PLC inhibitor D609 (10 μM) and PARP-1 inhibitor DPQ (25 μM)

Phospholipase C (PLC) has been reported to trigger PARP-1 activation independent of DNA damage [31]. Since PLC can be activated by TNFα receptor stimulation [43], we evaluated the possiblity that PLC mediates TNFα-induced PARP-1 activation by using inhibitors to block phopha tidyl choline-specific PLC (PC-PLC)-mediated phopha tidyl choline cleavage to diacyl glycerol (DAG) (D609; 10 μM) [44] and phosphatidyl inositol - specific PLC (PI-PLC)-mediated phosphatidyl inositol cleavage to IP_3_ (U-73112; 10 μM) [45, 46]. These studies showed that TNFα-induced PAR formation was blocked in cultures treated with D609, but not U-73112 (Fig. 3). Conversely, the PLC activator m-3M3FBS [47] mimicked effects of TNFα stimulation by inducing PARP-1 activation, NF-κB transcriptional activation, and microglial morphological transformation, and these effects of m-3M3FBS were likewise blocked by D609 (Fig. 4). These results suggest that the signaling pathway linking TNFα to PARP-1 activation involves activation of PC-PLC.

**Fig. 5:**
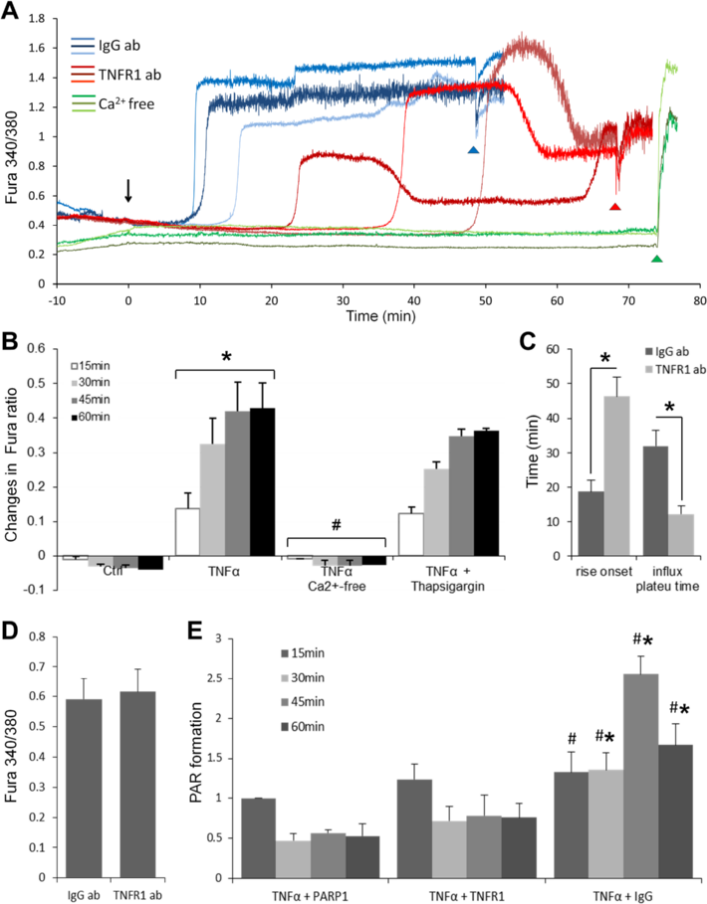
TNFα induces calcium influx via TNFα receptor 1 (TNFR1). **a** TNFα induced increase in intracellular free Ca^2+^, as indicated by the change in Fura2 emission. This increase was blocked in Ca^2+^-free medium (0 Ca^2+^ + 0.5 mM EGTA, *green*), and the intracellular calcium response profile was changed by neutralizing TNFR1 antibody (TNFR1 ab, *red*). IgG antibody (IgG ab, *blue*) was used as a control. *Arrow* denotes addition of TNFα (15 ng/ml) to the astrocyte cultures. The Ca^2+^ ionophore, A23187-Br (10 μM) was added at the end of each experiment (*arrow head*) to provide a calibration point. **b** TNFα-induced intracellular free Ca^2+^ (Fura2 ratio) increase was prevented in Ca^2+^-free medium but was not affected in cells pre-treated with thapsigargin (2 μM). ^*^ *p* < 0.05 compared to control at each time point; ^#^ *p* < 0.05 compared to TNFα at each designated time point, *n* = 5–6. **c**, **d** TNFR1 neutralizing antibody (TNFR1 ab) delayed calcium influx rise onset and reduced the influx plateau time, but the calcium influx maximum of the level of intracellular free Ca^2+^ (Fura2 ratio) was not affected between TNFR1 ab and IgG ab-pre-treated TNFα-stimulated cells. ^*^ *p* < 0.05, *n* = 5. **e** PARP-1 activation profile detected by PAR formation was also changed when TNFR1 was blocked. TNFR1 neutralizing antibody reduced the length and level of PAR formation, mimicking effects of PARP inhibitor. ^*^ *p* < 0.05, *n* = 3

TNFα receptor activation also induces calcium influx in astrocytes [48] and was confirmed by our studies (Fig. 5). TNFα-induced PARP-1 activation was blocked in calcium-free medium and by chelation of intracellular calcium with BAPTA-AM (Fig. 3). By contrast, pre-incubation with thapsigargin to deplete endoplastic reticulum (ER) calcium stores did not prevent either TNFα-induced intracellular Ca^2+^ elevations (Fig. 5b) or PAR formation (Fig. 3).

Studies with a TNF receptor 1 (TNFR1)-neutralizing antibody were performed to assess the role of this receptor subtype in PARP-1 activation. The neutralizing antibody delayed and reduced TNFα-induced intracellular Ca^2+^ elevations (Fig. 5a, c, d). The TNFR1 neutralizing antibody also reduced PAR formation (Fig. 5e), suggesting that TNFR1 has a critical role in PARP-1-related inflammatory responses.

### ERK2 inhibition prevents TNFα-induced PARP-1 activation

**Fig. 6:**
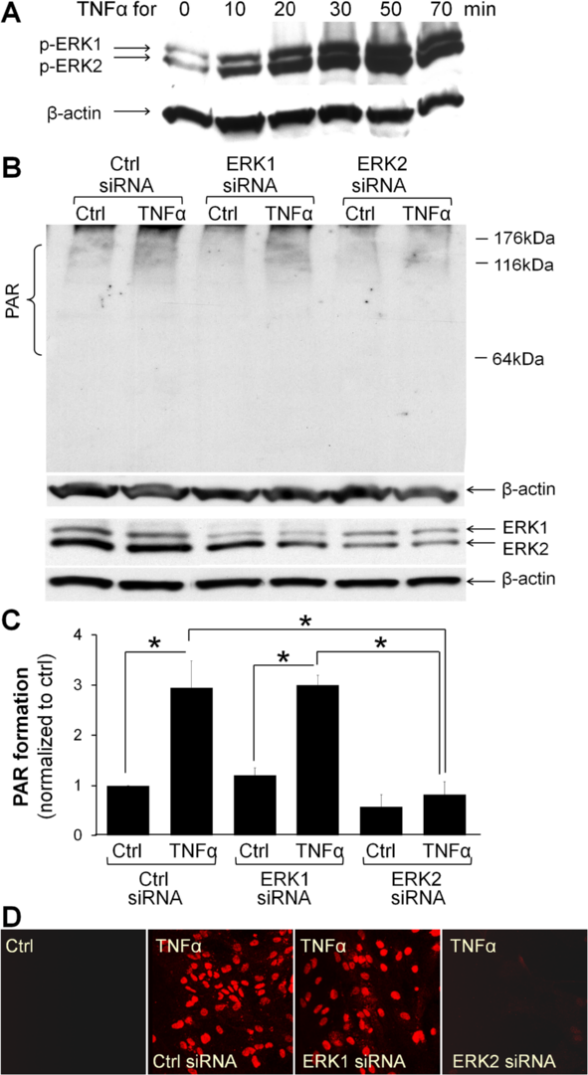
TNFα-induced PARP activation is regulated by ERK2. **a** TNFα (15 ng/ml) induces ERK1/2 phosphorylation in astrocyte cultures. Representative of *n* = 3. **b** PAR immunoblots shows that TNFα-induced PAR formation is reduced after siRNA downregulation of ERK2, but not ERK1. PAR is seen as a discrete band at 116 kDa attributable to auto-poly(ADP)-ribosylation of PARP-1 and as a smear over other molecular weights resulting from PAR formation on other proteins. Actin band shows protein loading. ERK1 and ERK2 expression are shown *below* the PAR western blot. The siRNA treatments reduced their expression by 72 ± 8 % and 72 ± 5 %, respectively, with protein loading shown by the actin band. **c** Quantification of PAR western blots, conditions as in **b**; * *p* < 0.05, *n* = 3. **d** Immunostaining for PAR also shows that TNFα-induced PAR formation is suppressed by siRNA downregulation of ERK2 expression. Images are representative of four independent experiments. All PAR formation studies are conducted 60 min after TNFα stimuli

ERK2 promotes PARP-1 activation by phosphorylating PARP-1 [35, 49]. The MEK/ERK kinase cascade is activated PC-PLC [50], thus suggesting ERK2 as a potential link between TNFα receptor activation and PARP-1 activation. Here, ERK1/2 phosphorylation (activation) began within 10 min of TNFα exposure (Fig. 6a, c). Inhibition of the ERK1/2 activation with PD98059 [51] prevented TNFα-induced PARP-1 activation in both astrocyte and microglia cultures (Fig. 3). siRNA reduction of ERK2 expression also prevented TNFα-induced PARP-1 activation, whereas ERK1 silencing had no effect (Fig. 6b, c, d).

### ERK2 phosphorylation of PARP-1 induces NF-κB activation

**Fig. 7:**
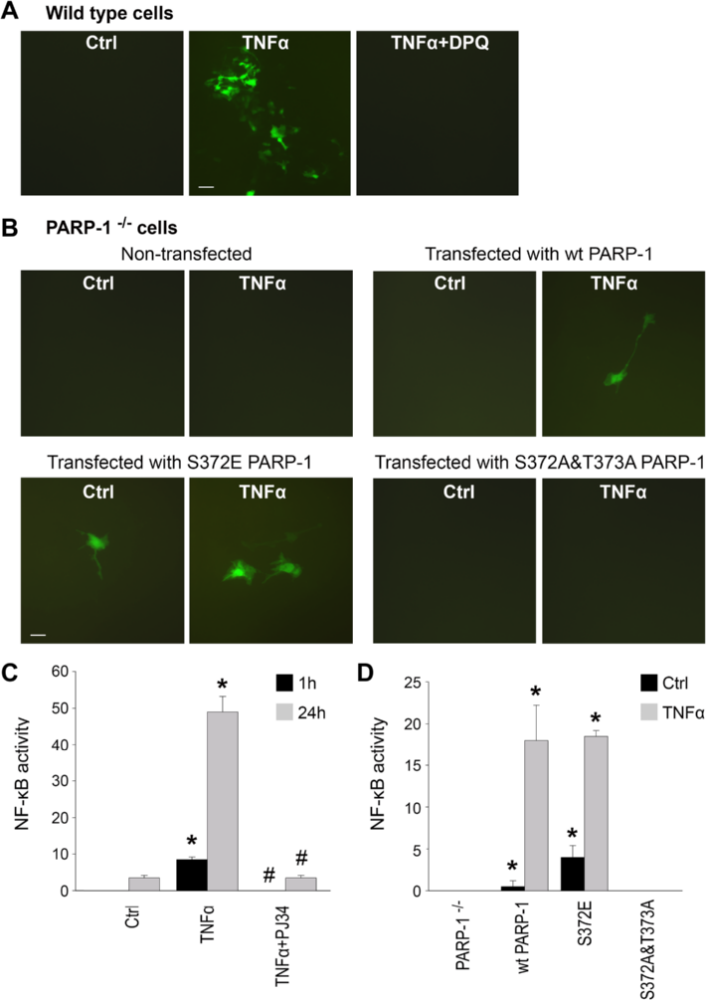
ERK2-mediated phosphorylation of PARP-1 induces NF-κB transcriptional activity. **a** NF-κB transcriptional activity was evaluated in astrocytes transfected with a reporter gene in which dEGFP expression (*green*) is driven by NF-κB p65 subunit binding. TNFα-induced (15 ng/ml). dEGFP expression is blocked by the PARP-1 inhibitor, DPQ (25 μM). *Scale bar*, 20 μm. Quantified data are shown in **c**. (*p < 0.05 compared to control, ^#^ *p* < 0.05 compared to TNFα, *n* = 3–4). **b** TNFα-induced GFP expression is absent in PARP1^−/−^ astrocytes and reconstituted in PARP-1^−/−^ astrocytes transfected with normal human PARP-1 (wt PARP-1) but not with mutant S372A and T372A PARP-1, which lacks ERK2 phosphorylation sites. Conversely, PARP-1^−/−^ astrocytes transfected with mutant S372E PARP-1, which has a phosphomimetic glutamate residue at an ERK2 phosphorylation site, show increased basal NF-κB transcriptional activation. *Scale bar*, 10 μm. Quantified data are shown in **d**. (*p < 0.05 compared to wt PARP-1, *n* = 3). Expression levels of the mutant PARP-1 species were comparable, as evaluated by PARP-1 western blots using a polyclonal antibody (not shown)

**Fig. 8:**
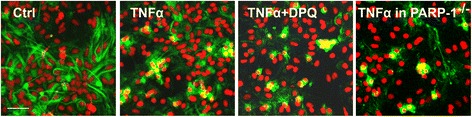
PARP-1 is not required for NF-κB nuclear translocation. The subcellular localization of the NF-κB p65 subunit (*green*) was visualized by immunostaining in wt and PARP-1^−/−^ astrocyte cultures after TNFα exposure. Nuclei were stained with propidium iodide (*red*). Cells fixed after 30 min of TNFα (15 ng/ml) exposure show overlap of these signals (*yellow*), indicative of p65 movement into the nucleus. This movement was not blocked by the PARP inhibitor DPQ (25 μM) or in PARP-1^−/−^ cells. Representative of *n* = 3. *Scale bar*, 50 μm

The role of ERK2-mediated phosphorylation in PARP-1 activation was further evaluated using PARP-1 constructs containing mutations at the ERK2 phosphorylation sites, S372 and T373 [35]. The S372A and T373A mutations prevent ERK2-mediated phosphorylation, and S372E provides a constitutive phosphorylation mimic. PARP-1^−/−^ astrocyte cultures were transfected with either wt or mutant PARP-1 constructs, and NF-κB transcriptional activation was assessed using a lentivirus-delivery of dEGFP reporter gene that is activated by the p65 subunit of NF-κB [36]. In cells transfected with wt PARP-1, TNFα induced a robust activation of the reporter gene within 1 h and a further increase at 24 h. This TNFα-induced transcriptional activation was absent in non-transfected cells. It was also absent in transfected cells treated with the PARP inhibitor DPQ, thus confirming a requirement for PARP-1 enzymatic activity in this process. Cells transfected with the mutant PARP-1 lacking ERK2 phosphorylation sites (S372A and T373A) also showed no TNFα-induced NF-κB activity. By contrast, cells transfected with the S372E PARP-1 construct, which mimics constitutive phosphorylation by ERK2, showed increased GFP expression in the absence of TNFα stimulation (Fig. 7). Of note, neither PARP-1 deficiency nor PARP-1 inhibition blocked the nuclear translocation of p65 induced by TNFα (Fig. 8). We also analyzed TNFα-induced cytokine release and saw decrease in NF-κB-dependent pro-inflammatory cytokines (IFNγ, IL-1β, KC, MCP-1, MIP-1β) in both PARP-1^−/−^ microglia and in wt microglia upon PARP inhibition with PJ34 (500 nM) (Table 1), providing more functional outcome measure for PARP-1 mediated NF-κB regulation. Interestingly, PARP-1 inhibition did not reduce anti-inflammatory cytokines (IL-4, IL-10, IL-13, TGFβ) (Table 1).

**Table 1:** Microglial cytokine and trophic factor release in vitro upon TNFα stimulation

	Pro-inflammatory cytokines				
	IFNγ	IL-1β	KC	MCP-1	MIP-1β
Ctrl (wt)	221.97 ± 36.69	20.4 ± 1.9	157.12 ± 20.8	103.63 ± 21.5	2840.02 ± 357.4
TNFα (wt)	335.76 ± 21.3^*^	59.38 ± 2.8^*^	408.13 ± 60.0^*^	668.0 ± 78.7^*^	4777.28 ± 527.6^*^
TNFα + PJ34 (wt)	284.09 ± 21.6^**^	35.2 ± 2.3^**^	197.75 ± 25.4^**^	339.35 ± 77.9^**^	3081.38 ± 353.7^**^
PJ34 (wt)	68.48 ± 16.6^*^	1.35 ± 2.0^*^	24.67 ± 11.1^*^	153.96 ± 33.4	1664.42 ± 361.8^*^
Ctrl (PARP-1^−/−^)	101.03 ± 21.2^*^	13.82 ± 4.0^*^	22.30 ± 6.7^*^	46.84 ± 16.7^*^	1104.99 ± 172.0^*^
TNFα (PARP-1^−/−^)	101.97 ± 17.9^**^	14.12 ± 3.0^**^	67.07 ± 18.9^**^	84.10 ± 28.7^**^	1118.34 ± 146.1^**^
	Anti-inflammatory cytokines and trophic factors				
	IL-4	IL-10	IL-13	TGF-β	VEGF
Ctrl (wt)	0.8 ± 0.5	21.1 ± 1.2	1.03 ± 0.1	1.11 ± 0.2	4.280 ± 1.2
TNFα (wt)	1.46 ± 0.5	22.78 ± 10.9	0.2 ± 0.3^*^	1.35 ± 0.2	2.82 ± 0.6
TNFα + PJ34 (wt)	6.09 ± 0.8^*^	34.77 ± 8.3^*^	25.00 ± 4.8^**^	1.41 ± 0.1	39.57 ± 6.6^**^
PJ34 (wt)	1.48 ± 0.4	19.0 ± 1.3	0.89 ± 0.2	1.31 ± 0.1^*^	22.92 ± 3.7^*^
Ctrl (PARP-1^−/−^)	0.77 ± 0.1	7.47 ± 11.3	5.73 ± 1.1^*^	1.37 ± 0.1^*^	13.63 ± 5.3^*^
TNFα (PARP-1^−/−^)	0.86 ± 0.4	11.16 ± 1.9	2.69 ± 0.5^**^	1.90 ± 0.2^**^	21.98 ± 4.7^**^

Data is presented as a pg of cytokine detected from the cell culture medium, normalized to mg of protein in corresponding cell culture well (mean ± stdev). PJ34 is a PARP-1 inhibitor. ^*^ *p* ≤ 0.05 compared to ctrl (wt), ^**^ *p* ≤ 0.05 compared to TNFα (wt), *n* = 3–4

### PLC does not have a role in PARP-1 activation induced by DNA damage

**Fig. 9:**
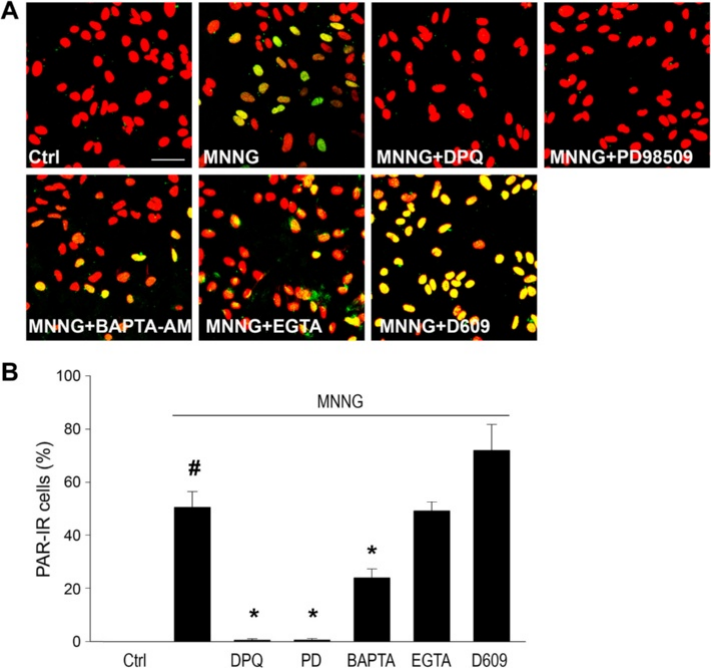
PLC is not required for DNA damage-induced PARP-1 activation. **a** Astrocytes were immunostaining for PAR (*green*) and nuclei were counterstained with propidium iodide (*red*). PAR formation induced by MNNG (10 μM, 60 min) was prevented by inhibitors of PARP (DPQ; 25 μM) and MEK-ERK1/2 (PD98059; 10 μM), but not by PC-PLC inhibitor (D609; 10 μM) or by calcium-free medium (500 μM EGTA). Intracellular calcium chelation with BAPTA-AM (2 μM) produced a partial reduction in PAR formation. *Scale bar*, 40 μm. **b** Quantification of PAR positive cells (^#^ *p* < 0.05 compared to control, *p < 0.05 compared to MNNG alone, *n* = 3)

Taken together, these findings suggest a signaling pathway in which TNFα interaction with TNFR1 triggers prolonged Ca^2+^ influx, PC-PLC activation, ERK2 phosphorylation, and activation of PARP-1. We therefore evaluated whether this pathway is involved in PARP-1 activation induced by DNA damage. Astrocytes were exposed to 10 μM MNNG to induce DNA damage sufficient to produce PARP-1 activation comparable to that induced by TNFα (Fig. 2c). DNA damage-induced PARP-1 activation, like TNFα-induced activation, was blocked by the MEK/ERK inhibitor PD9089; however, it was not blocked by the PLC inhibitor D609 or by Ca^2+^-free medium and was only partly blocked by BAPTA-AM (Fig. 9).

## Discussion

**Fig. 10:**
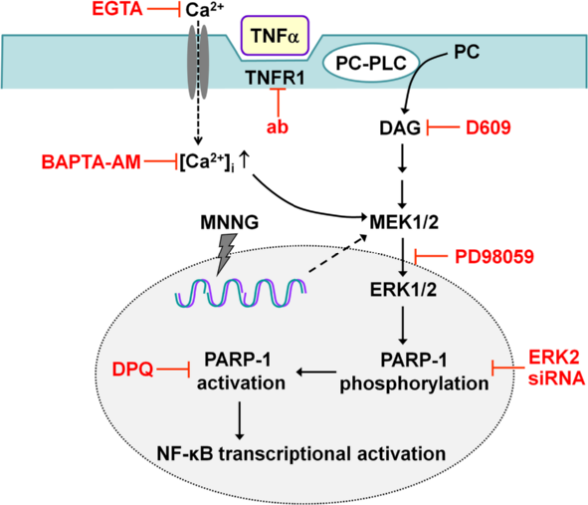
The schematic summary of TNFα-induced PARP-1 activation pathway in glial cells. PARP-1 activation induced by TNFα does not involve DNA damage, which is a classical inducer of PARP-1 activation. A MNNG-induced DNA damage activation pathway differs from a TNFα-induced one, but they both share the requirement of ERK2-mediated PARP-1 phosphorylation. TNFα binding to its receptor TNFR1 induces calcium influx, and activates phosphotidyl choline-specific phospholipase (PC-PLC) leading to phosphotidyl choline cleavage to diacyl glycerol (DAG). This process leads to activation of mitogen-activated protein kinase pathway (MEK1/2-ERK1/2) and ERK2-mediated phosphorylation of PARP-1, which allow PARP-1 activation. Enzymatically active PARP-1 co-activates NF-κB transcriptional activation promoting pro-inflammatory secretion profile. TNFα-induced PARP-1 activation was prevented by calcium chelators (EGTA, BAPTA-AM), TNFR1-neutralizing antibody (ab), inhibitors of PC-PLC-DAG (D609), MEK1/2-ERK1/2 (PD98059), PARP (DPQ) and ERK2 siRNA, while MNNG-induced PARP activation was affected only by inhibitors of ERK2 and PARP. TNFα-induced NF-κB transcription activation was prevented by inhibitors of PC-PLC-DAG, ERK1/2 and PARP

The mechanism by which PARP-1 is activated by TNFα and mediates TNFα pro-inflammatory effects has not been previously established. Results of these studies suggest that TNFα induces PARP-1 activation by a pathway involving TNFR1, calcium entry, activation of PC-PLC, and activation of the MEK1/ERK2 protein kinase cascade. This signaling pathway differs in key respects from the pathway involved in classical PARP-1 activation by DNA damage (Fig. 10). These results also show that PARP-1 enzymatic activity is required for TNFα-induced NF-κB transcriptional activation and related cytokine production in glia.

NF-κB activation involves its translocation to the nucleus, transcription complex formation and binding to DNA [2]. However, these events alone do not necessarily result in NF-κB-driven gene transcription; transcription of the target gene can be prevented by parallel binding of repressors or other contradicting processes [12, 52, 53]. Results presented here with an NF-κB reporter gene assay provide an example of this; PARP-1 inhibitors allowed NF-κB translocation to the nuclei, but blocked NF-κB-mediated gene transcription of pro-inflammatory cytokine, while some anti-inflammatory cytokines (IL-10, IL-13), which have a κB site in their promoter [54, 55] were not reduced by PARP-1 ablation. The reporter gene approach also clarifies the importance of PARP-1 enzymatic activity in NF-κB activation. Several reports indicate that PARP-1 inhibitors can block glial inflammatory responses and the associated upregulation of proteins with NF-κB promotor sites [11, 13–15, 18, 56], but other reports suggest that PARP-1 enzymatic activity is not required for NF-κB transcriptional activation [24]. The present results demonstrate that PARP-1 enzymatic activity is required for transcriptional activity induced by the p65 subunit of NF-κB in astrocytes. However, we cannot exclude a non-enzymatic interaction between PARP-1 and other NF-κB DNA-binding subunits or in non-canonical NF-κB activation [57]. Both genetic depletion and pharmacologic inhibition of PARP-1 were found to inhibit NF-κB-dependent pro-inflammatory cytokines, but interestingly, anti-inflammatory cytokines were not inhibited. This suggests that immune modulation ability of PARP-1 [37] is a result of complex transcriptional regulation, possibly involving other transcription factors [58].

Although PARP-1 activation is classically associated with DNA damage, in which PARP-1 binding to DNA strand breaks or kinks triggers its enzymatic activation, PARP-1 activation also occurs in settings such as gene transcription and neurotrophin signaling, in which DNA damage is not known to occur [25–31]. PARP-1 activation induced by TNFα would similarly be expected to occur independent of DNA damage, but it is also possible that TNFα could upregulate pro-oxidant inflammatory responses and thereby lead to DNA damage [59]. This possibility was excluded here using the PANT assay, which showed no increase in single-strand DNA breaks in cultures incubated with TNFα.

Our results indicate some commonalities in the signal transduction pathways mediating DNA damage-induced PARP-1 activation and TNFα-induced PARP-1 activation. PARP-1 activation induced by DNA damage requires PARP-1 to be phosphorylated at an ERK2 phosphorylation site [35]. Here, PARP-1 activation induced by TNFα was found to be blocked by siRNA downregulation of ERK2 expression and by PD98059, which inhibits ERK2 activation. In addition, cells expressing mutant PARP-1 that lack the ERK2 phosphorylation at sites S372 and T373 showed no PARP-1 activation [35] and no NF-κB transcriptional activation, whereas cells expressing mutant PARP-1 containing the phosphomimetic S372E mutation showed an increase in both basal PARP-1 activity and basal NF-κB transcriptional activity. These findings suggest that ERK2 phosphorylation of PARP-1 at S372 and/or T373 is required for PARP-1 activation triggered by either DNA damage or by TNFα. We cannot exclude the possibility that other mitogen-activated protein kinases (MAPKs) have a role in TNFα-induced PARP-1 activation. TNFα certainly stimulates phosphorylation of p38 MAPK and cJun N-terminal kinase (JNK) [60], which could at least indirectly facilitate PARP-1 activation or parallel processes. Indeed, activation of JNK is a required downstream event in PARP-1-mediated (MNNG-induced) necrotic cell death [61, 62], and p38 interaction with PARP-1 is a crucial step in hydrogen peroxide-induced cell death pathway [63]. Notably, phosphorylation of both of these MAPKs were identified downstream of PARP-1 activation. Our previous studies assessed the role of p38, JNK, and ERK1/2 in MNNG-induced PARP-1 activation and cell death, and only ERK1/2 inhibition reduced both [35].

Our results also indicate differences in the DNA damage-induced and TNFα-induced PARP-1 activation pathways. PARP-1 activation by MNNG, a DNA alkylating agent, was not inhibited by the PC-PLC inhibitor D609 or by Ca^2+^-free medium and only partially inhibited by the intracellular Ca^2+^ chelator BAPTA-AM. By contrast, TNFα-induced PARP-1 activation was completely blocked by D609, Ca^2+^-free medium, and BAPTA-AM. The Ca^2+^_i_ increase induced by TNFα was abrogated by Ca^2+^-free medium but not by thapsigargin pre-treatment, suggesting that the increased Ca^2+^_i_ originates from the extracellular space rather than the ER. Also arguing against a role for ER calcium release was the finding that TNFα-induced PARP-1 activation was not blocked by U73112, an inhibitor of PI-PLC-induced IP_3_ formation, but was inhibited by D609, which inhibits PC-PLC-induced DAG formation. Similarly, neutralizing antibody against TNFR1, extracellular receptor for TNFα, significantly diminished the TNFα-induced increase in Ca^2+^_i_ and PAR formation. Moreover, the PLC-activating compound m-3M3FBS induced PARP-1 activation and NF-κB transcription activity, which were prevented by inhibitor targeting PLC-DAG signaling in the absence of other stimuli, suggesting that this pathway is sufficient to induce PARP-1 activity independent of DNA damage. These findings are congruent with prior work; TNFα binding to its TNFR1 receptor can stimulate PC-PLC activation and production of DAG (as reviewed by [50]. DAG precedes PKC activation, which in turn activates the MEK/ERK protein kinase cascade in astocytes [64]. In macrophage cultures, LPS-induced activation was reported to involve PC-PLC, DAG, PKCζ, and ERK1/2 [65]. While PKCs are likely mediators between DAG and MEK/ERK activation also in the signaling pathway described in our present report, further studies are required to identify whether they have a role in PARP-1 activation and which subunits are involved. The potential route for calcium influx could be through store-operated channels (SOCs), which are known to be regulated by TNFα-induced K^+^ current upon TNFα stimulation in human microglia [66]. PLC-DAG activation has also been reported to trigger opening of SOCs [67, 68].

Our data demonstrate the critical role of TNFR1 in PARP-1-related inflammatory responses, which is fitting as TNFR1 is a dominant pro-inflammatory receptor expressed by microglia and also associated with NF-κB-dependent anti-apoptotic transcriptional activity [69]. However, we cannot completely exclude the role of TNFR2, as the data relies on specificity of TNFR1 antibody, and there is no commercially available neutralizing antibody for TNFR2. It also remains to be established how the described signaling components (i.e., PC-PLC, calcium influx, and ERK2) upon TNFR1 stimulation interact with the classical TNFR adaptor proteins sucs as TRADD, TRAF-2, and RIP1.

A key role for ERK2 in DNA damage-independent PARP-1 activation has been previously attributed to a non-enzymatic interaction between ERK2 and PARP-1 [30, 31]. Prior studies have also suggested a role for PLC-mediated liberation of IP_3_ and ER calcium from within the nucleus itself as a factor stimulating PARP-1 activity [31]. The present findings do not address this possibility directly, but the failure of thapsigargin or D609 to block PARP-1 activation, along with the suppression of PARP-1 activation in Ca^2+^-free medium, argues against ER Ca^2+^ release as a major factor in the setting of TNFα stimulation. Whether these experimental differences are attributable to cell-type differences, differing cell stimuli, or other factors remains to be established.

PARP-1 is by far the most abundant and active PARP species in the cell nucleus, but PARP-2 and PARP-3 are also present [40, 70, 71]. Recent findings have identified distinct roles for PARP-2 and PARP-3 in the astrocyte inflammatory responses induced by *S. Aureus* [72]. A major contribution of PARP-2 or PARP-3 to the PAR formation and NF-κB activation observed in the present studies is unlikely because no PAR formation or NF-κB activation was observed in PARP-1 deficient cells. However, the differing PARP species interact [73, 74]. It is therefore possible that the different PARP species and interactions between these species may have signal-specific effects on transcription factor activities.

## Conclusions

TNFα stimulation of glial cells leads to PARP-1 activation via a mechanism that is independent of DNA strand breaks. TNFα stimulates PARP-1 activation by a pathway involving TNFR1, calcium entry, activation of PC-PLC, and activation of the MEK1/ERK2 protein kinase cascade. PARP-1 enzymatic activity is required for TNFα-induced NF-κB transcriptional activation and pro-inflammatory cytokine release.

## Abbreviations

BSS: balanced salt solution
Ca^2+^: calcium
DAG: diacylglycerol
ERK: extracellular signal-regulated kinase
FBS: fetal bovine serum
IFNγ: interferon gamma
IL: interleukin
IP_3_: inositol trisphosphate
KC: keratinocyte-derived cytokine
MCP: monocyte chemoatractant protein
MEK: mitogen-activated protein kinase kinase
MEM: modified Eagle’s medium
MIP: macrophage inflammatory protein
MNNG: N-methyl-N'-nitro-N-nitrosoguanidine
NF-κB: nuclear factor kappa B
PANT: DNA-polymerase I-mediated biotin-dATP nick translation
PAR: poly(ADP-ribose)
PARP-1: poly(ADP-ribose) polymerase-1
PC: phophotidyl choline
PLC: phosphol lipase C
TGFβ: transforming growth factor beta
TNFα: tumor necrosis factor alpha
TNFR: tumor necrosis factor receptor

## References

[1] Shohami E, Ginis I, Hallenbeck JM (1999). Dual role of tumor necrosis factor alpha in brain injury. Cytokine Growth Factor Rev.

[2] Ghosh S, Hayden MS (2008). New regulators of NF-kappaB in inflammation. Nature reviews. Immunology.

[3] Yan P, Liu N, Kim GM, Xu J, Li Q, Hsu CY (2003). Expression of the type 1 and type 2 receptors for tumor necrosis factor after traumatic spinal cord injury in adult rats. Exp Neurol.

[4] Kahn MA, Dopp JM, Liva S, MacKenzie-Graham AJ, Chang R, Huang A (1999). Temporal kinetics and cellular phenotype of TNF p55/p75 receptors in experimental allergic encephalomyelitis. J Neuroimmunol.

[5] Basak S, Hoffmann A (2008). Crosstalk via the NF-kappaB signaling system. Cytokine Growth Factor Rev.

[6] Ullrich O, Diestel A, Eyupoglu IY, Nitsch R (2001). Regulation of microglial expression of integrins by poly(ADP-ribose) polymerase-1. Nat Cell Biol.

[7] Erdelyi K, Bakondi E, Gergely P, Szabo C, Virag L (2005). Pathophysiologic role of oxidative stress-induced poly(ADP-ribose) polymerase-1 activation: focus on cell death and transcriptional regulation. Cell Mol Life Sci.

[8] Hassa PO, Hottiger MO (2002). The functional role of poly(ADP-ribose)polymerase 1 as novel coactivator of NF-kappaB in inflammatory disorders. Cell Mol Life Sci.

[9] Gagne JP, Isabelle M, Lo KS, Bourassa S, Hendzel MJ, Dawson VL (2008). Proteome-wide identification of poly(ADP-ribose) binding proteins and poly(ADP-ribose)-associated protein complexes. Nucleic Acids Res.

[10] Rouleau M, Aubin RA, Poirier GG (2004). Poly(ADP-ribosyl)ated chromatin domains: access granted. J Cell Sci.

[11] Nakajima H, Nagaso H, Kakui N, Ishikawa M, Hiranuma T, Hoshiko S (2004). Critical role of the automodification of poly(ADP-ribose) polymerase-1 in nuclear factor-kappaB-dependent gene expression in primary cultured mouse glial cells. J Biol Chem.

[12] Chang WJ, Alvarez-Gonzalez R (2001). The sequence-specific DNA binding of NF-kappa B is reversibly regulated by the automodification reaction of poly (ADP-ribose) polymerase 1. J Biol Chem.

[13] Chiarugi A, Moskowitz MA (2003). Poly(ADP-ribose) polymerase-1 activity promotes NF-kappaB-driven transcription and microglial activation: implication for neurodegenerative disorders. J Neurochem.

[14] Kauppinen TM, Swanson RA (2005). Poly(ADP-ribose) polymerase-1 promotes microglial activation, proliferation, and matrix metalloproteinase-9-mediated neuron death. J Immunol.

[15] Kauppinen TM, Higashi Y, Suh SW, Escartin C, Nagasawa K, Swanson RA (2008). Zinc triggers microglial activation. J Neurosci Off J Soc Neurosci.

[16] Hasko G, Mabley JG, Nemeth ZH, Pacher P, Deitch EA, Szabo C (2002). Poly(ADP-ribose) polymerase is a regulator of chemokine production: relevance for the pathogenesis of shock and inflammation. Mol Med.

[17] Haddad M, Rhinn H, Bloquel C, Coqueran B, Szabo C, Plotkine M (2006). Anti-inflammatory effects of PJ34, a poly(ADP-ribose) polymerase inhibitor, in transient focal cerebral ischemia in mice. Br J Pharmacol.

[18] Ha HC, Hester LD, Snyder SH (2002). Poly(ADP-ribose) polymerase-1 dependence of stress-induced transcription factors and associated gene expression in glia. Proc Natl Acad Sci U S A.

[19] Oliver FJ, Menissier-de Murcia J, Nacci C, Decker P, Andriantsitohaina R, Muller S (1999). Resistance to endotoxic shock as a consequence of defective NF-kappaB activation in poly (ADP-ribose) polymerase-1 deficient mice. Embo J.

[20] Zingarelli B, Salzman AL, Szabo C (1998). Genetic disruption of poly (ADP-ribose) synthetase inhibits the expression of P-selectin and intercellular adhesion molecule-1 in myocardial ischemia/reperfusion injury. Circ Res.

[21] Le Page C, Sanceau J, Drapier JC, Wietzerbin J (1998). Inhibitors of ADP-ribosylation impair inducible nitric oxide synthase gene transcription through inhibition of NF kappa B activation. Biochem Biophys Res Commun.

[22] Schreiber V, Dantzer F, Ame JC, de Murcia G (2006). Poly(ADP-ribose): novel functions for an old molecule. Nat Rev Mol Cell Biol.

[23] Kraus WL, Lis JT (2003). PARP goes transcription. Cell.

[24] Hassa PO, Covic M, Hasan S, Imhof R, Hottiger MO (2001). The enzymatic and DNA binding activity of PARP-1 are not required for NF-kappa B coactivator function. The Journal of biological chemistry.

[25] Tulin A, Spradling A (2003). Chromatin loosening by poly(ADP)-ribose polymerase (PARP) at Drosophila puff loci. Science.

[26] Petermann E, Keil C, Oei SL (2005). Importance of poly(ADP-ribose) polymerases in the regulation of DNA-dependent processes. Cell Mol Life Sci.

[27] Chang P, Jacobson MK, Mitchison TJ (2004). Poly(ADP-ribose) is required for spindle assembly and structure. Nature.

[28] Visochek L, Steingart RA, Vulih-Shultzman I, Klein R, Priel E, Gozes I (2005). PolyADP-ribosylation is involved in neurotrophic activity. J Neurosci.

[29] Cohen-Armon M, Visochek L, Katzoff A, Levitan D, Susswein AJ, Klein R (2004). Long-term memory requires polyADP-ribosylation. Science.

[30] Cohen-Armon M, Visochek L, Rozensal D, Kalal A, Geistrikh I, Klein R (2007). DNA-independent PARP-1 activation by phosphorylated ERK2 increases Elk1 activity: a link to histone acetylation. Mol Cell.

[31] Homburg S, Visochek L, Moran N, Dantzer F, Priel E, Asculai E (2000). A fast signal-induced activation of Poly(ADP-ribose) polymerase: a novel downstream target of phospholipase c. J Cell Biol.

[32] Wang ZQ, Auer B, Stingl L, Berghammer H, Haidacher D, Schweiger M (1995). Mice lacking ADPRT and poly(ADP-ribosyl)ation develop normally but are susceptible to skin disease. Genes Dev.

[33] Swanson RA, Farrell K, Stein BA (1997). Astrocyte energetics, function, and death under conditions of incomplete ischemia: a mechanism of glial death in the penumbra. Glia.

[34] Chen J, Jin K, Chen M, Pei W, Kawaguchi K, Greenberg DA (1997). Early detection of DNA strand breaks in the brain after transient focal ischemia: implications for the role of DNA damage in apoptosis and neuronal cell death. J Neurochem.

[35] Kauppinen TM, Chan WY, Suh SW, Wiggins AK, Huang EJ, Swanson RA (2006). Direct phosphorylation and regulation of poly(ADP-ribose) polymerase-1 by extracellular signal-regulated kinases 1/2. Proc Natl Acad Sci U S A.

[36] Chen J, Zhou Y, Mueller-Steiner S, Chen LF, Kwon H, Yi S (2005). SIRT1 protects against microglia-dependent amyloid-beta toxicity through inhibiting NF-kappaB signaling. J Biol Chem.

[37] Kauppinen TM, Suh SW, Higashi Y, Berman AE, Escartin C, Won SJ (2011). Poly(ADP-ribose)polymerase-1 modulates microglial responses to amyloid beta. J Neuroinflammation.

[38] Smith PK, Krohn RI, Hermanson GT, Mallia AK, Gartner FH, Provenzano MD (1985). Measurement of protein using bicinchoninic acid. Anal Biochem.

[39] Virag L, Szabo C (2002). The therapeutic potential of poly(ADP-ribose) polymerase inhibitors. Pharmacol Rev.

[40] Fisher AE, Hochegger H, Takeda S, Caldecott KW (2007). Poly(ADP-ribose) polymerase 1 accelerates single-strand break repair in concert with poly(ADP-ribose) glycohydrolase. Mol Cell Biol.

[41] Oliver FJ, Menissier-de Murcia J, de Murcia G (1999). Poly(ADP-ribose) polymerase in the cellular response to DNA damage, apoptosis, and disease. Am J Hum Genet.

[42] Molinete M, Vermeulen W, Burkle A, Menissier-de Murcia J, Kupper JH, Hoeijmakers JH (1993). Overproduction of the poly(ADP-ribose) polymerase DNA-binding domain blocks alkylation-induced DNA repair synthesis in mammalian cells. Embo J.

[43] Machleidt T, Kramer B, Adam D, Neumann B, Schutze S, Wiegmann K (1996). Function of the p55 tumor necrosis factor receptor “death domain” mediated by phosphatidylcholine-specific phospholipase C. J Exp Med.

[44] Kiss Z, Tomono M (1995). Compound D609 inhibits phorbol ester-stimulated phospholipase D activity and phospholipase C-mediated phosphatidylethanolamine hydrolysis. Biochim Biophys Acta.

[45] Smith RJ, Sam LM, Justen JM, Bundy GL, Bala GA, Bleasdale JE (1990). Receptor-coupled signal transduction in human polymorphonuclear neutrophils: effects of a novel inhibitor of phospholipase C-dependent processes on cell responsiveness. J Pharmacol Exp Ther.

[46] Moreno-Garcia ME, Lopez-Bojorques LN, Zentella A, Humphries LA, Rawlings DJ, Santos-Argumedo L (2005). CD38 signaling regulates B lymphocyte activation via a phospholipase C (PLC)-gamma 2-independent, protein kinase C, phosphatidylcholine-PLC, and phospholipase D-dependent signaling cascade. J Immunol.

[47] Bae YS, Lee TG, Park JC, Hur JH, Kim Y, Heo K (2003). Identification of a compound that directly stimulates phospholipase C activity. Mol Pharmacol.

[48] Koller H, Thiem K, Siebler M (1996). Tumour necrosis factor-alpha increases intracellular Ca2+ and induces a depolarization in cultured astroglial cells. Brain.

[49] Gagne JP, Moreel X, Gagne P, Labelle Y, Droit A, Chevalier-Pare M (2009). Proteomic investigation of phosphorylation sites in poly(ADP-ribose) polymerase-1 and poly(ADP-ribose) glycohydrolase. J Proteome Res.

[50] Schutze S, Machleidt T, Kronke M (1994). The role of diacylglycerol and ceramide in tumor necrosis factor and interleukin-1 signal transduction. J Leukoc Biol.

[51] Dudley DT, Pang L, Decker SJ, Bridges AJ, Saltiel AR (1995). A synthetic inhibitor of the mitogen-activated protein kinase cascade. Proc Natl Acad Sci U S A.

[52] Dong J, Jimi E, Zhong H, Hayden MS, Ghosh S (2008). Repression of gene expression by unphosphorylated NF-kappaB p65 through epigenetic mechanisms. Genes Dev.

[53] Kauppinen TM, Gan L, Swanson RA (2013). Poly(ADP-ribose) polymerase-1-induced NAD(+) depletion promotes nuclear factor-kappaB transcriptional activity by preventing p65 de-acetylation. Biochim Biophys Acta.

[54] Hinz M, Lemke P, Anagnostopoulos I, Hacker C, Krappmann D, Mathas S (2002). Nuclear factor kappaB-dependent gene expression profiling of Hodgkin’s disease tumor cells, pathogenetic significance, and link to constitutive signal transducer and activator of transcription 5a activity. J Exp Med.

[55] Xu LG, Shu HB (2002). TNFR-associated factor-3 is associated with BAFF-R and negatively regulates BAFF-R-mediated NF-kappa B activation and IL-10 production. J Immunol.

[56] Martinez-Zamudio RI, Ha HC (2014). PARP1 enhances inflammatory cytokine expression by alteration of promoter chromatin structure in microglia. Brain and behavior.

[57] Neumann M, Naumann M (2007). Beyond IkappaBs: alternative regulation of NF-kappaB activity. Faseb J.

[58] Kraus WL, Hottiger MO (2013). PARP-1 and gene regulation: progress and puzzles. Mol Asp Med.

[59] Ullrich O, Diestel A, Bechmann I, Homberg M, Grune T, Hass R (2001). Turnover of oxidatively damaged nuclear proteins in BV-2 microglial cells is linked to their activation state by poly-ADP-ribose polymerase. Faseb J.

[60] Kant S, Swat W, Zhang S, Zhang ZY, Neel BG, Flavell RA (2011). TNF-stimulated MAP kinase activation mediated by a Rho family GTPase signaling pathway. Genes Dev.

[61] Xu Y, Huang S, Liu ZG, Han J (2006). Poly(ADP-ribose) polymerase-1 signaling to mitochondria in necrotic cell death requires RIP1/TRAF2-mediated JNK1 activation. J Biol Chem.

[62] Douglas DL, Baines CP (2014). PARP1-mediated necrosis is dependent on parallel JNK and Ca(2)(+)/calpain pathways. J Cell Sci.

[63] Robaszkiewicz A, Valko Z, Kovacs K, Hegedus C, Bakondi E, Bai P (2014). The role of p38 signaling and poly(ADP-ribosyl)ation-induced metabolic collapse in the osteogenic differentiation-coupled cell death pathway. Free Radic Biol Med.

[64] Abe K, Saito H (2000). The p44/42 mitogen-activated protein kinase cascade is involved in the induction and maintenance of astrocyte stellation mediated by protein kinase C. Neurosci Res.

[65] Cuschieri J, Billgren J, Maier RV (2006). Phosphatidylcholine-specific phospholipase C (PC-PLC) is required for LPS-mediated macrophage activation through CD14. J Leukoc Biol.

[66] McLarnon JG, Franciosi S, Wang X, Bae JH, Choi HB, Kim SU (2001). Acute actions of tumor necrosis factor-alpha on intracellular Ca(2+) and K(+) currents in human microglia. Neuroscience.

[67] Putney JW, Trebak M, Vazquez G, Wedel B, Bird GS (2004). Signalling mechanisms for TRPC3 channels. Novartis Found Symp.

[68] Vig M, Kinet JP (2009). Calcium signaling in immune cells. Nat Immunol.

[69] Probert L (2015). TNF and its receptors in the CNS: The essential, the desirable and the deleterious effects. Neuroscience.

[70] Ame JC, Spenlehauer C, de Murcia G (2004). The PARP superfamily. Bioessays.

[71] Rouleau M, El-Alfy M, Levesque MH, Poirier GG (2009). Assessment of PARP-3 distribution in tissues of cynomolgous monkeys. J Histochem Cytochem.

[72] Phulwani NK, Kielian T (2008). Poly (ADP-ribose) polymerases (PARPs) 1–3 regulate astrocyte activation. J Neurochem.

[73] Huber A, Bai P, de Murcia JM, de Murcia G (2004). PARP-1, PARP-2 and ATM in the DNA damage response: functional synergy in mouse development. DNA repair.

[74] Loseva O, Jemth AS, Bryant HE, Schuler H, Lehtio L, Karlberg T (2010). PARP-3 is a mono-ADP-ribosylase that activates PARP-1 in the absence of DNA. J Biol Chem.

